# Carcinogenic and non-carcinogenic health risks associated with heavy metal exposure in commonly consumed marine fish in Egypt

**DOI:** 10.1038/s41598-026-67201-w

**Published:** 2026-08-22

**Authors:** Hend Ali Elshebrawy, Khalid Ibrahim Sallam, Nahed Gomaa Kasem, Huping Xue, Fatma A. El-Gohary

**Affiliations:** 1 Department of Food Hygiene, Safety, and Technology, Faculty of Veterinary Medicine, Mansoura University, Mansoura, 35516 Egypt; 2 College of Animal Science and Technology, Northwest A&F University, Yangling, Shaanxi China; 3 Department of Hygiene and Zoonoses, Faculty of Veterinary Medicine, Mansoura University, Mansoura, 35516 Egypt

**Keywords:** Heavy metals, Consumed marketed fish, Carcinogenic, Non-carcinogenic indices, EDI, THQ, CR, Environmental sciences, Risk factors

## Abstract

This study aimed to quantify the levels of mercury (Hg), arsenic (As), lead (Pb), and cadmium (Cd) in 250 fish samples, including flathead grey mullet, sardine, and mackerel, collected from local fish markets in Mansoura, Egypt, on ten occasions from August 2023 to March 2024. Unlike previous studies that have primarily focused on metal concentrations, this study assessed both carcinogenic and non-carcinogenic health risks among the general population (57.09 g/day) and high-fish consumers (200 g/day). Metal concentrations were determined using atomic absorption spectrophotometry, with method accuracy validated using certified reference materials (CRMs), yielding recoveries of 95.5–100.6%. The average concentrations of Hg, As, Pb, and Cd (µg/g wet weight) were 0.313, 2.13, 0.194, and 0.018 in flathead grey mullet (As > Hg > Pb > Cd); 0.131, 1.65, 0.472, and 0.021 in sardine (As > Pb > Hg > Cd); and 0.196, 0.98, 0.370, and 0.025 in mackerel (As > Pb > Hg > Cd), respectively. Hg and As concentrations were significantly higher in flathead grey mullet, whereas Pb concentration was highest in sardine and Cd concentration was highest in mackerel. Based on the Maximum Permissible Limits (MPLs) set by the Food and Agriculture Organization (FAO), Egyptian Organization for Standardization and Quality (EOS), and Food Standards Australia New Zealand (FSANZ), the proportion of samples exceeding these limits ranged from 9 to 20% for Hg, 22–56% for As, and 20–58% for Pb. For the general population, only the estimated daily intake (EDI) of Hg in flathead grey mullet was above the Provisional Tolerable Daily Intakes (PTDIs), while EDI of most metals exceeded safety thresholds in high fish consumers. Target Hazard Quotient (THQ) for Hg and As exceeded 1.0, while TTHQ values for flathead grey mullet, sardine, and mackerel were 8.4, 5.7, and 4.35 for the general population and 29.43, 19.84, and 15.26 for high-fish consumers, respectively, indicating potential non-carcinogenic health risks. Furthermore, cancer risk (CR) for As ranged from 1.20E−03 to 9.14E−03, exceeding the acceptable safety threshold of 1.0E−04, whereas Pb and Cd risks remained within safe limits. The relatively high metal concentrations may reflect anthropogenic pollution and other environmental inputs affecting the aquatic ecosystem, highlighting the need for regular monitoring and control of pollution sources to reduce contamination and protect public health.

## Introduction

Fish is a nutritious and valuable food, rich in easily digestible, high-quality protein, vitamins, minerals, and polyunsaturated fatty acids (PUFAs), particularly omega-3 fatty acids, which support cardiovascular and brain health, immunity, and overall growth^1^. In Egypt, fish is widely consumed across all socioeconomic groups as a healthy, flavorful, and affordable source of animal protein. Egypt’s per capita fish consumption was approximately 20.84 kg in 2022, ranking fifth in Africa^2^.

Egypt is considered a leading fish-producing country owing to its distinctive geographical location at the northeastern corner of Africa, with a coastline of around 2500 km on both the Mediterranean Sea and Red Sea, in addition to several lakes (Manzala, Bardawil, Burullus, Mariout, and others), and the Nile River with its tributaries^3,4^. Although Egypt ranks among Africa’s top fish producers, imports are still required to satisfy demand. Recent economic pressures, such as rising inflation and currency shortages, have pushed fish prices higher, causing a slight reduction in per capita consumption^5^.

In Egypt, marine and coastal fisheries play an important role in the local seafood supply, particularly in coastal cities such as Alexandria, Port Said, and Damietta^3^. Among the commonly consumed marine fish species in Egypt are flathead grey mullet (*Mugil cephalus*), locally known as Bouri, sardine (*Sardinella aurita*), and mackerel (*Scomber scombrus*) because of their affordability and nutritional value, as they are rich in high-quality protein, minerals, and omega-3 PUFAs^6–8^. Nevertheless, marine fish can accumulate heavy metals from contaminated aquatic environments, potentially increasing human exposure through fish consumption. The extent of heavy metal accumulation may vary among fish species depending on factors such as fish size, diet, water quality, habitat, and other ecological characteristics^7^.

The flathead grey mullet is a cosmopolitan, omnivorous species with coastal-pelagic habits, predominantly inhabiting estuaries, lagoons, and shallow coastal waters at depths of less than 20 meters^7,9^. Sardine is a small pelagic fish, distributed mainly in tropical and subtropical seas, including the Mediterranean Sea^7^. Sardine is a plankton-feeding fish that has a vital role in the marine food web through transferring energy and nutrients from planktonic organisms to higher predators^10^. Mackerel is also a commonly consumed pelagic fish in Egypt, reaching the Egyptian market through frozen imports and fresh catches from fisheries along the Mediterranean and Red Sea coasts^6^.

Heavy metals (HMs) are persistent metallic elements of high atomic weight and density that can enter aquatic environments through both natural processes, such as dissolution from the Earth’s crust, and anthropogenic activities, including agriculture, industrial processes, urbanization, sewage disposal, and surface runoff^11–14^. HMs include both toxic elements such as mercury (Hg), arsenic (As), lead (Pb), and cadmium (Cd), as well as essential metals such as copper, zinc, and iron, which are indispensable for human health but can be toxic above their threshold levels^15^. The Agency for Toxic Substances and Disease Registry (ATSDR) listed Hg, As, Pb, and Cd among the top ten most hazardous substances, highlighting their potential health hazards^16^. The bioaccumulation of HMs in fish tissues may be affected by various factors, including the fish’s size, age, sex, living environment, feeding behavior, and swimming patterns^17^. Additionally, seasonal variations and water quality can influence metal accumulation and associated health risks in aquatic organisms^18^. Furthermore, sediment characteristics can act as important drivers of heavy metal bioaccumulation in fish and associated human health risks^19,20^.

From a public health perspective, consuming contaminated marine fish with HMs beyond recommended levels could increase the risk of neurological damage, renal disease, liver disease, and other serious conditions^21^. Mercury, particularly methylmercury, is highly toxic to the nervous system, while arsenic is ranked first among the top ten most hazardous substances by ATSDR^16^. Arsenic is a highly toxic element; however, more than 90% of As in marine fish occurs in organic forms, which are less toxic, whereas only 0.02–11% is inorganic As, which is highly toxic^22^. Inorganic As could cause cardiovascular dysfunction, skin disorders, neurological injury, liver damage, and malignancy on long-term exposure^23^. Lead exposure may impair hemoglobin synthesis and cause neurological, renal, and reproductive disorders, whereas cadmium (Cd) is classified as a Group 1 human carcinogen by the International Agency for Research on Cancer (IARC). Moreover, the ingestion of foods contaminated with cadmium can disrupt vital physiological functions and cause nephrotoxicity, hepatotoxicity, and cardiovascular disorders^23,24^.

Exposure to Hg, As, Pb, and Cd has become a growing public health concern; therefore, several international and national organizations have established maximum permissible limits (MPLs) for these contaminants. Previous studies in Egypt have evaluated the health risks of consuming HMs by estimating the Estimated Daily Intakes (EDIs) and comparing them with safety thresholds, such as the Provisional Tolerable Daily Intake (PTDI) and Benchmark Dose Level (BMDL) established by regulatory organizations, such as the Joint FAO/WHO Expert Committee on Food Additives (JECFA) and the European Food Safety Authority (EFSA)^3,6–8,25^. Health risk assessment generally considers both non-carcinogenic and carcinogenic effects. Non-carcinogenic risk refers to the potential for adverse health effects other than cancer and is commonly evaluated using the target hazard quotient (THQ) and total target hazard quotient (TTHQ), whereas carcinogenic risk represents the estimated lifetime probability of developing cancer and is assessed using the cancer risk (CR) index^26,27^.

Mansoura is a major Egyptian city, where marine fish, including flathead grey mullet, sardine, and mackerel, are widely marketed and consumed. Compared with previous Egyptian studies, the present study focuses on commercially available marine fish collected from retail markets in Mansoura and considers both general and high-fish consumption scenarios. Therefore, this study aimed to determine the concentrations of mercury (Hg), arsenic (As), lead (Pb), and cadmium (Cd) in flathead grey mullet, sardine, and mackerel marketed in Mansoura, Egypt, using atomic absorption spectrophotometry (AAS), with the analytical method validated for linearity, precision, recovery, and detection and quantification limits, and to assess potential health risks using the estimated daily intake (EDI), target hazard quotient (THQ), total target hazard quotient (TTHQ), and cancer risk (CR(.

## Materials and methods

### Study area

**Fig. 1:**
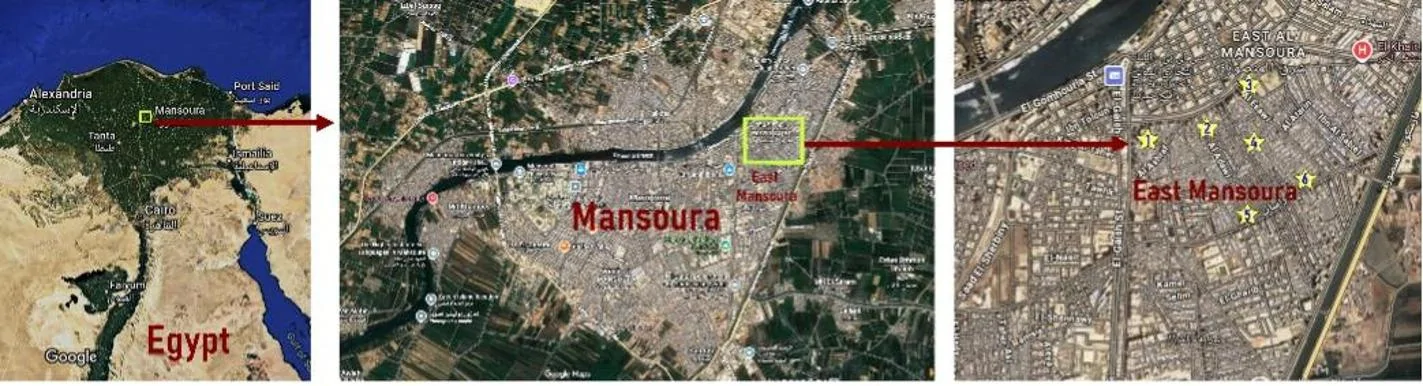
Map of the sampling areas for the fish analyzed in the present study. Satellite basemap imagery was obtained from Google Maps (Google LLC; https://www.google.com/maps/). Labels, sampling-site coordinates, arrows, and other graphical elements were added by the authors using Microsoft PowerPoint (Version 2021; Microsoft Corporation).

Fish samples were collected from local fish markets in Mansoura City, Egypt (Fig. 1). Mansoura lies on the Nile’s east bank, approximately 120 km northeast of Cairo^28^. The city serves as a major commercial hub with high fish consumption. Fish markets in Mansoura provide a wide variety of marine fish, either from the Egyptian Mediterranean coastal fisheries, mainly Kafr El Sheikh, El Bahira, Damietta, and Port Said governorates, or imported frozen fish. The mackerel samples (*Scomber scombrus*) were commercially available imported frozen fish, primarily sourced from Norway and the Netherlands, according to vendors’ information.

### Sample collection

**Fig. 2:**
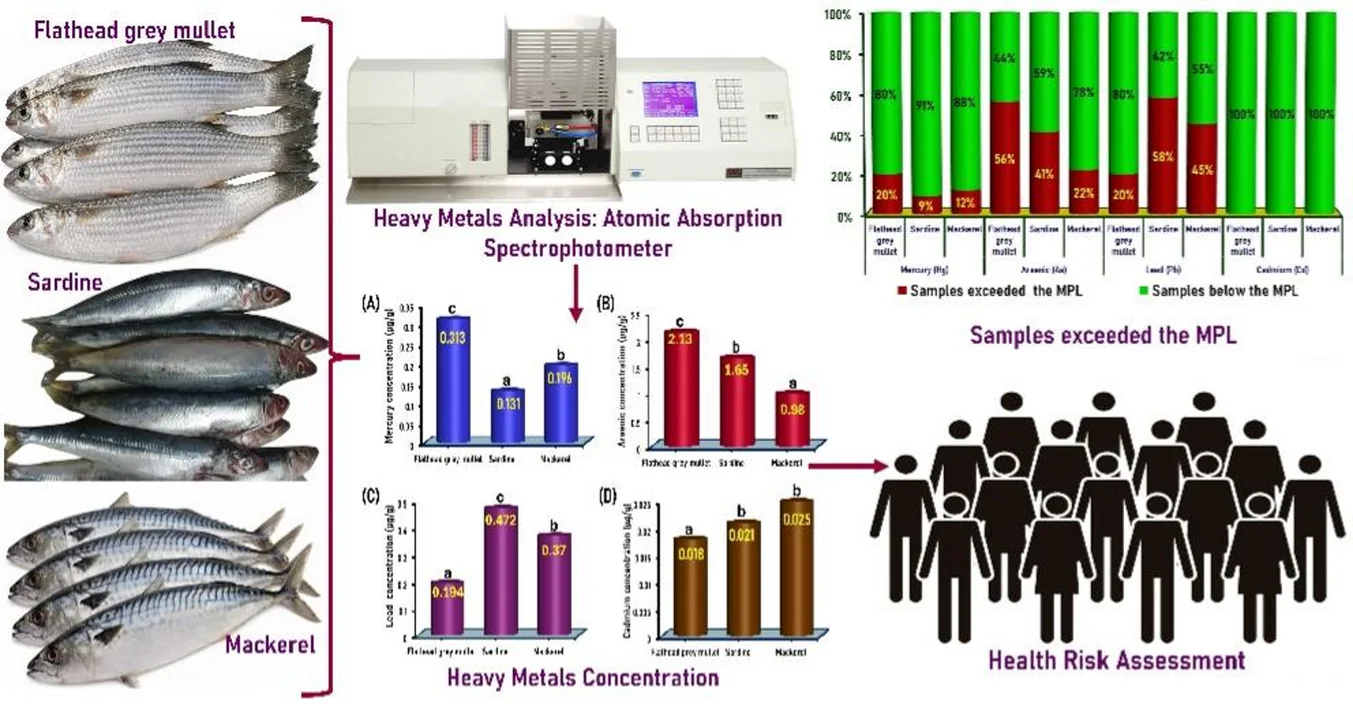
A graphical abstract of the study design, workflow, and results.

A total of 250 fish samples, including 50 flathead grey mullet (*Mugil cephalus*) and 100 each of sardine (*Sardinella aurita*) and mackerel (*Scomber scombrus*) with different lengths and weights, were collected from six retail fish outlets in East Mansoura City, Egypt (Fig. 1), across ten sampling occasions between August 2023 and March 2024. During each sampling occasion, five outlets were randomly selected from which one mullet, two sardines, and two mackerel were purchased per outlet. Total body length ranged from 31.0 to 43.0 cm for flathead grey mullet, 10.7 to 14.0 cm for sardine, and 27.0 to 39.0 cm for mackerel. Body weight ranged from 332.0 to 485.0 g for flathead grey mullet, 19.0 to 43.0 g for sardine, and 394.0 to 517.0 g for mackerel. Each sample was individually placed in an impermeable polyethylene bag, placed in an icebox (4 °C), and immediately transported to the Department of Food Hygiene, Safety, and Technology, Faculty of Veterinary Medicine, Mansoura University, Egypt. At the laboratory, each sample was weighed by a digital balance, transferred to a clean polyethylene bag, then labeled with the collection date and identification number, and kept at -20 °C till HMs are analyzed. As the study involved only commercially available fish samples obtained from retail markets and did not involve human participants or live animal experimentation, ethical approval for human or animal subjects was not applicable. The study was conducted in accordance with the research integrity and ethical guidelines of Mansoura University. The graphical abstract summarizing the plan of the current study is presented in Fig. 2.

### Chemicals and laboratory wares preparation

All chemicals used were of ultrapure grade (Merck KGaA, Darmstadt, Germany), including perchloric acid (70%), nitric acid (65%), hydrochloric acid (37%), and hydrogen peroxide (30%). All laboratory wares used for fish sample digestion, handling, and storage were thoroughly cleaned by soaking in soap and water for at least 2 h, followed by several rinses with running tap water. The wares were then sequentially rinsed once with deionized water, once with Therands mixture [250 ml deionized water + 200 ml concentrated HCl (37%) + 80 ml H₂O₂ (30%)], once with washing acid [900 ml deionized water + 100 ml concentrated HCl (37%)], and finally with deionized water, following the procedure described by Sallam et al.^29^, after cleaning, the wares were dried in an incubator until use.

### Sample digestion

Fish tissue samples were individually digested following the wet digestion technique previously outlined by Sallam et al.^29^. In brief, 2 g portions of dorsal muscle along with the overlying skin from each selected sample were excised aseptically with a sterile stainless-steel scalpel and forceps, macerated using a ceramic knife, and put into a screw-capped tube containing 8 ml of nitric acid (65%) and 4 ml of perchloric acid (70%). To ensure laboratory safety and allow gas release during acid reaction, the tubes were loosely covered and incubated in a water bath at 53 °C overnight. This procedure resulted in a clear, colorless digest with no visible residual organic matter. All tubes were then allowed to cool to room temperature. The cooled digest was then diluted with deionized water and filtered through a Whatman filter number 42 (Merck, Darmstadt, Germany) into clean glass beakers. The filtrate was subsequently diluted with deionized water to a final volume of 50 ml. The resultant filtrates were placed into clean, screw-capped bottles, labeled with the fish species and sample number, and stored at room temperature until HMs analysis. Blank solutions were prepared following the same wet digestion procedure, but without adding samples to detect any background contamination from reagents or equipment. To guarantee the accuracy of HMs determination, any detected residues were deducted from the final results.

### Analysis of HMs

Heavy metals (Hg, As, Pb, and Cd) were quantified using an Atomic Absorption Spectrophotometer (AAS) (Buck Scientific 210 VGP, Inc.) at the Central Laboratory, Faculty of Veterinary Medicine, Zagazig University, Egypt, following the method described by AOAC^30^. Hg and As were analysed by a flameless AAS equipped with a hydride generation system for As and a cold vapor system for Hg. Meanwhile, Pb and Cd were analysed by flame AAS with an oxidizing air–acetylene flame. The apparatus was set to detect Hg, As, Pb, and Cd at wavelengths of 253.7 nm, 193.7 nm, 283.3 nm, and 228.8 nm, respectively. The detection limits (µg/g) for these metals.

HM concentrations were expressed as µg/g wet weight (ppm). They were obtained from the digital readout of the AAS and calculated using Eq. (1):

1 $$C=~\frac{{R \times D}}{W}$$

where C represents the heavy metal concentration (µg/g wet weight), R is the reading (ppm) from the digital scale of AAS, D is the dilution factor of the measured sample, and W is the sample weight (g).

In addition, blank sample readings were measured and subtracted from all sample measurements to determine any potential contamination.

### Validation of analytical methods for HM determination in fish samples

The validation of analytical parameters used for HM quantification in fish samples, including calibration curves’ linearity, limit of detection (LOD), and limit of quantification (LOQ), instrumental precision, spike recovery %, and precision of certified reference material (CRM), is presented in Table 1.

**Table 1:** Validation of instrumental, digestion, and Certified Reference Material (CRM) precision for heavy metal analysis in fish samples.

	Precision criteria**	Heavy metals concentrations (µg/g)			
		Hg	As	Pb	Cd
Instrumental precision	LOD	0.017	0.194	0.132	0.010
	LOQ	0.056	0.640	0.436	0.033
	Precision (CV %)	2.25	2.84	2.12	0.79
	Mean of spiked amount *	0.375 ± 0.03	1.50 ± 0.04	0.500 ± 0.02	0.25 ± 0.03
	Mean of recovered amount *	0.362 ± 0.02	1.52 ± 0.03	0.494 ± 0.02	0.243 ± 0.02
	Mean of spike recovery %	96.5%	101.3%	98.8%	97.2%
Calibration curve	Standard solution concentrations (mg/L) for the calibration curve***	0.005, 0.01, 0.05, 0.1, 0.5, 2.0, 5.0	0.01, 0.05, 0.1, 0.5, 1.0, 5.0, 20.0	0.005, 0.01, 0.05, 0.25, 0.5, 5, 10	0.005, 0.01, 0.05, 0.25, 0.5, 2.0, 5
	Correlation coefficient (R^2^)	0.999	0.999	0.999	0.999
Precision (RSD %) of the digestion method for heavy metal determination in fish matrix (*n* = 5).	Concentration of metal in fish sample	0.05 ± 0.01	2.69 ± 0.07	0.47 ± 0.03	0.04 ± 0.01
	Amount of metal added	0.15	5.0	2.0	0.15
	Concentration of metal in the spiked sample	0.196 ± 0.002	7.62 ± 0.09	2.38 ± 0.04	0.185 ± 0.004
	Recovery (%)	97.3	98.6	95.5	96.7
	RSD (%)	2.28	2.64	3.75	4.83
Certified reference material (CRM)**** validation	Certified value ^a^	0.44 ± 0.18	34.6 ± 2.4	0.162 ± 0.032	14.5 ± 0.6
	Observed value ^b^	0.42 ± 0.03	34.3 ± 0.8	0.163 ± 0.01	14.1 ± 0.2
	Recovery %	95.5	99.1	100.6	97.2

* Data are presented as mean ± standard error (SE) of triplicate measurements.

**LOD, limit of detection; LOQ, limit of quantification; RSD, relative standard deviation; CRM, certified reference material; SE, standard error; R², correlation coefficient.

*** Calibration standard concentrations were prepared according to AOAC Official Method 2015.01^31^.

**** DOLT-5: Dogfish Liver Certified Reference Material for Trace Metals and other Constituents-from the National Research Council of Canada (NRC-CNRC). https://nrc-digital-repository.canada.ca/eng/view/crt/?id=78bfab6b-21cf-4733-a871-cac430f711f5&dsl=en.

^a^ Certified values are given as mean ± expanded uncertainty.

^b^ Observed value represented by Mean ± SE; (*n* = 5).

To prepare the working standards, stock standard solutions (1000 mg/L) of Hg, As, Pb, and Cd were diluted with acidified ultrapure water (5% v/v HNO₃) according to AOAC Official Method^31^. Calibration curves were constructed using standard concentrations of 0.005, 0.01, 0.05, 0.1, 0.5, 2.0, and 5.0 mg/L for Hg; 0.01, 0.05, 0.1, 0.5, 1.0, 5.0, and 20.0 mg/L for As; 0.005, 0.01, 0.05, 0.25, 0.5, 5, and 10 mg/L for Pb; and 0.005, 0.01, 0.05, 0.25, 0.5, 2.0, and 5.0 mg/L for Cd. The correlation coefficient (R²) for each calibration curve was 0.999, indicating excellent linearity.

For the instrumental precision, LOD and LOQ values were calculated using Eq. 2:

2 $$X=~F~ \times \frac{{SD}}{b}$$

where X represents either LOD or LOQ, F represents the factor of 3.3 for LOD and 10 for LOQ; SD: Standard deviation of the blank; b: Slope of the regression line.

To assess the digestion precision, fish samples were spiked with known concentrations of each metal (0.15 µg/g for Hg and Cd, 5 µg/g for As, and 2 µg/g for Pb) and analyzed following the same digestion and analytical procedures. The recovery rates for spiked samples were 97.3%, 98.6%, 95.5%, and 96.7% for Hg, As, Pb, and Cd, respectively (Table 1).

The accuracy of all analytical procedures was verified using DOLT-5, Dogfish liver CRM from the National Research Council of Canada (NRC-CNRC). The CRM recovery percentages ranged from 95.5% to 100.6% (Table 1).

### Health risk assessment

#### Estimated daily intake (EDI)

The Estimated Daily Intake (EDI) of the analyzed HMs was calculated for both the general population and high fish consumers following Eq. (3), outlined by USEPA^32^.

3 $$EDI=\frac{{{{\mathrm{C}}_{\mathrm{m}}} \times {\mathrm{IR}}}}{{BW}}$$

EDI is the estimated daily intake (µg/g bw/day). C_m_ is the HM concentration in the sample and is expressed as µg/g wet weight. IR is the average fish consumption per day, which is 57.09 g/d for the general population^2^ and 200 g/d for high-fish consumers^33^. BW is the average body weight of Egyptian fish consumers (16–70 years), which equals 70 kg.

The EDIs were calculated and compared with their provisional tolerable daily intakes (PTDI) or benchmark dose levels (BMDL). The JECFA set the PTDI for MeHg and the BMDL for As at 2.30E−04 µg/g bw/day and 3.00E−03 µg/g bw/day, respectively^34^, while EFSA recommended the BMDL for Pb (6.30E−04 µg/g bw/day)^35^, and JECFA set the PTDI for Cd (8.30E−04 µg/g bw/day)^36^.

#### Assessment of Non-carcinogenic health risks using THQ and TTHQ

THQ and TTHQ are parameters used to assess the potential non-carcinogenic health risk linked to lifetime exposure to toxic metals from consuming fish. The THQ was determined using Eq. (4), previously described by USEPA^32^.

4 *THQ=EDI/RfD*

where EDI refers to the estimated daily intake of HMs (µg/g/day), RfD (the oral reference dose) represents the estimated daily amount of contaminant that a person can ingest over a lifetime without causing any non-carcinogenic health risks. The RfD for Hg and that for As are 0.0001 and 0.0003, respectively^26^, while the RfD for Pb is 0.004^37,38^ and that for Cd is 0.001^39^.

Hazard Index (HI) or TTHQ was estimated using Eq. (5), endorsed by USEPA^32^.

5 $${\mathrm{TTHQ}}={{{\Sigma}}}TH{Q_{Hg}}+TH{Q_{As}}+TH{Q_{Pb}}+TH{Q_{Cd}}$$

THQ or TTHQ values > 1 indicate the potential for non-carcinogenic health risks associated with HM intake, with the risk increasing with higher values, whereas values below 1 reveal negligible risk to human health^26^.

#### Assessment of cancer risk (CR) in the general population and high-fish consumers

The probability of cancer occurrence over a lifetime because of the consumption of fish contaminated with HMs was calculated using Eq. (6), as described by the USEPA^26^.

6 *CR=CSF × EDI*

where CR represents the cancer risk, CSF refers to the ingestion cancer slope factor (µg/g /day), and EDI is the estimated daily intake of HMs (µg/g/day). No oral CSF has been established for Hg in fish. The CSF values are 1.5 µg/g /day for inorganic As^26^, whereas CSF values of 0.0085 and 0.38 µg/g/day were adopted from published literature for Pb and Cd, respectively^8,20,40^.

The CR values between 1E−4 and 1E−6 are acceptable, whereas values exceeding 1E−4 indicate a potential carcinogenicity^26^.

### Statistical analysis

All measurements were conducted in triplicate (analytical replicates), and the concentrations of HMs were expressed as means ± standard error (SE). Data were analyzed by one-way analysis of variance (ANOVA) to evaluate the differences in concentrations of HMs across all fish species examined. Mean differences were identified using Tukey’s honestly significant difference (HSD) test. Furthermore, Spearman’s rank correlation analysis was performed to evaluate the relationships between fish size parameters (total length and body weight) and heavy metal concentrations. Statistical significance was considered at *P* < 0.05 or *P* < 0.01. Data were analyzed by SPSS Statistics version 27.0 (IBM Corp., Armonk, NY, US).

## Results and discussions

### Concentrations of HMs in the muscle of flathead grey mullet, sardine, and mackerel

**Fig. 3:**
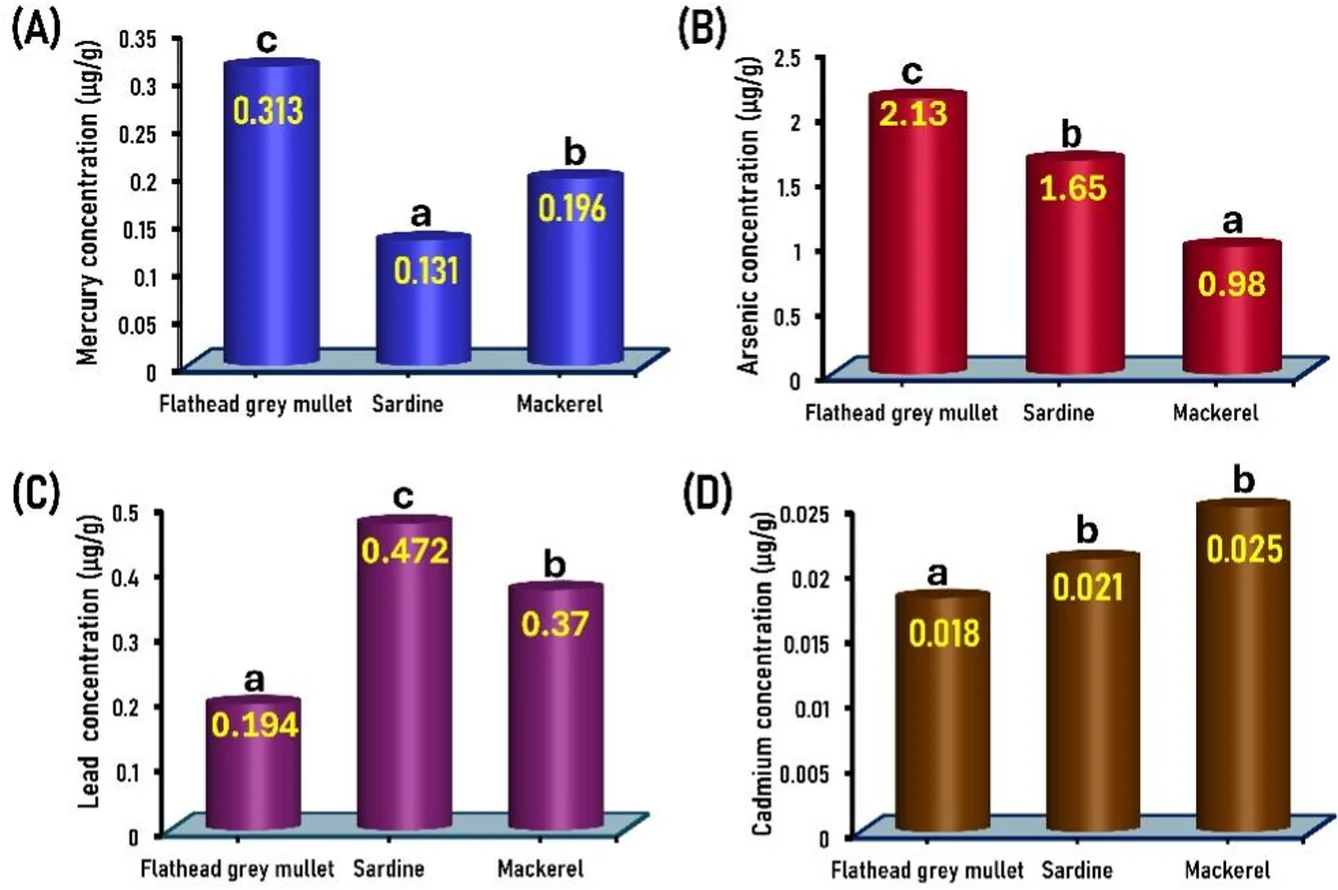
Mean concentrations of heavy metals (µg/g wet weight) in the muscle of flathead grey mullet, sardine, and mackerel. Columns with different letters for each metal indicate statistically significant differences at *P* < 0.05 or *P* < 0.01.

The mean concentrations of Hg, As, Pb, and Cd in muscle tissues of the three fish species examined are shown in Fig. 3. Arsenic (As) showed the highest mean level across all species. In contrast, Cd revealed the lowest mean concentration. Furthermore, the flathead grey mullet exhibited the highest concentrations of Hg and As, whereas sardine had the highest Pb concentration, and mackerel showed the highest Cd concentration (Fig. 3). Variations in metal levels among fish species may be influenced by differences in water quality, ecological factors, fish size, and dietary habits. This is in accordance with Abd-Elghany et al., who attributed differences in Hg concentrations among fish species to variations in feeding habits, habitat preferences, geographical distribution, and seasonal changes^25^. However, in the present study, Spearman’s rank correlation analysis revealed no statistically significant correlations (*P* > 0.05) between fish size parameters (total length and body weight) and the concentrations of Hg, As, Pb, and Cd across all fish species examined.

#### Concentrations of As

Mercury is a toxic HM that contaminates aquatic environments through industrial and agricultural discharges. Fish can accumulate Hg by consuming benthic organisms that ingest contaminated sediments. The Hg levels found in flathead grey mullet, sardine, and mackerel were in ranges of 0.01 to 0.66, 0.04 to 0.91, and 0.02 to 1.04 µg/g wet weight (Table 2), with mean values of 0.313 ± 0.02, 0.131 ± 0.04, and 0.196 ± 0.05 µg/g, respectively (Fig. 3A). Flathead grey mullet had a significantly (*P* < 0.05) higher mean Hg level, while sardine showed significantly lower mean Hg levels when compared with other species (Fig. 3A). In this context, Monier et al. mentioned that *Mugil cephalus* accumulates higher levels of HMs than other species because of their feeding habits, as they stay near sediment and feed on minute invertebrates, fish parts, algae, and detritus^7^. Our results are consistent with the study by El-Sherbiny and Sallam, who found that the mean Hg concentration in mackerel was significantly higher than that of sardine samples (0.413 µg/g versus 0.272 µg/g)^6^.

**Table 2:** Range of heavy metals in the muscles of flathead grey mullet, sardine, and mackerel, with comparison to maximum permissible limits (MPL).

Fish species	Metal analyzed	Range of metal analyzed (µg/g)		MPL (µg/g wet weight)	The percentage and number of samples exceeded the MPL
		Minimum level	Maximum level		
Flathead grey mullet	Hg	0.01	0.66	**0.5** ^**a, b**^	20% (10/50)
	As	0.54	5.60	**2.0** ^**c**^	56% (28/50)
	Pb	0.05	1.18	**0.3** ^**a, b**^	20% (10/50)
	Cd	0.01	0.04	**0.05** ^**b**^	0% (0/50)
Sardine	Hg	0.04	0.91	**0.5** ^**a, b**^	9% (9/100)
	As	0.35	4.19	**2.0** ^**c**^	41% (41/100)
	Pb	0.07	1.19	**0.3** ^**a, b**^	58% (58/100)
	Cd	0.01	0.07	**0.10** ^**b**^	0% (0/100)
Mackerel	Hg	0.02	1.04	**0.5** ^**a, b**^	12% (12/100)
	As	0.22	3.23	**2.0** ^**c**^	22% (22/100)
	Pb	0.03	1.25	**0.3** ^**a, b**^	45% (45/100)
	Cd	0.01	0.1	**0.10** ^**b**^	0% (0/100)

Bold values indicate the Maximum permissible limit (MPL).

^a^ FAO^43^.

^b^ Egyptian Organization for Standardization and Quality^44^.

^c^ Food Standards Australia New Zealand^45^.

#### Concentrations of As

Arsenic displayed the highest mean level among all HMs analyzed (Fig. 3B). The As concentrations in flathead grey mullet, sardine, and mackerel ranged from 0.54 to 5.60, 0.35 to 4.19, and 0.22 to 3.23 µg/g wet weight (Table 2), with mean values of 2.13 ± 0.2, 1.65 ± 0.1, and 0.98 ± 0.06 µg/g, respectively (Fig. 3B). Flathead grey mullet exhibited significantly higher As concentrations compared to sardine (*P* < 0.05) and mackerel (*P* < 0.01) (Fig. 3B). This can be attributed to its benthic feeding habits, as the species primarily consumes detritus, sediments, algae, and organic matter that accumulate higher arsenic levels^7^. In contrast, sardine is a plankton-feeding fish that transfers energy and nutrients from planktonic organisms to larger pelagic species^10^. This behavior may explain why sardine samples contain higher arsenic levels than mackerel (1.65 µg/g versus 0.98 µg/g) (Fig. 3B). Similarly, Bordajandi et al. found that As concentration in sardine was higher than that in mackerel (2.65 µg/g versus 0.91 µg/g)^41^.

#### Concentrations of lead

Lead is a highly toxic HM that poses significant risks to human health, making its accumulation in fish muscle an important concern for food safety. The Pb levels ranged from 0.05 to 1.18, 0.07 to 1.19, and 0.03 to 1.25 µg/g (Table 2), with mean levels of 0.194, 0.472, and 0.37 µg/g in the muscle of flathead grey mullet, sardine, and mackerel, respectively (Fig. 3C). The contamination level of Pb in fish species in the present study was in the following order: sardine > mackerel > flathead grey mullet (Fig. 3C). Sardine exhibited significantly higher Pb concentrations compared with mackerel (*P* < 0.05) and flathead grey mullet (*P* < 0.01) (Fig. 3C). These differences in metal concentrations among the three fish species examined might be related to the pollution level at the catching sites, feeding habits, the size of the caught fish, or complex interactions among biological and ecological factors^6^.

#### Concentrations of Cd

Cadmium residues were detected in the muscle of flathead grey mullet, sardine, and mackerel at ranges of 0.01 to 0.04, 0.01 to 0.07, and 0.01 to 0.1 µg/g with mean ± SE levels of 0.018 ± 0.003, 0.021 ± 0.002, and 0.025 ± 0.004 µg/g, respectively (Fig. 3D). The level of Cd contamination in fish species examined was in the following order: mackerel > sardine > flathead grey mullet (Fig. 3D). These variations in Cd concentrations may be due to their differences in detoxification capacity^42^. Flathead grey mullet and sardine likely sequester Cd more efficiently, via higher metallothionein activity, resulting in lower muscle concentrations. In contrast, mackerel appears to have comparatively lower detoxification efficiency with higher feeding intensity, leading to greater Cd retention. Cadmium had the lowest mean level among all HMs analyzed in our study (Fig. 3D). This may be attributed to its accumulation potential in the liver and kidney rather than in muscle tissues^42^.

### Comparison of HMs detected in fish muscles with their maximum permissible limit (MPL)

**Fig. 4:**
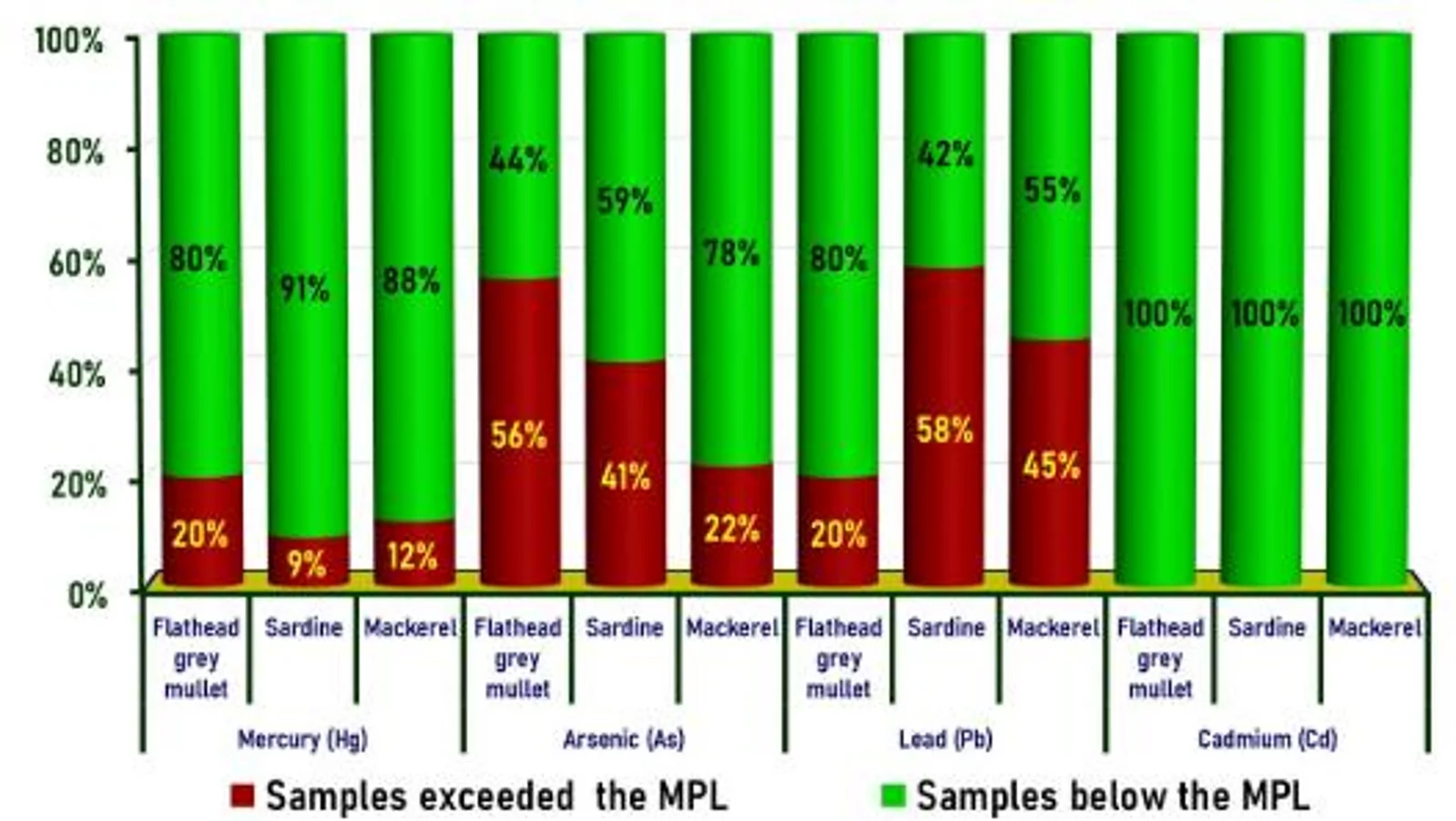
Percentage of fish samples exceeding or within the maximum permissible limits (MPLs) of Hg, As, Pb, and Cd in the muscle of flathead grey mullet, sardine, and mackerel.

HM concentrations in muscle samples were compared with their MPLs to assess the safety and acceptability of the fish species examined. For Hg, 20% (10/50), 9% (9/100), and 12% (12/100) of flathead grey mullet, sardine, and mackerel samples analyzed, respectively, exceeded the MPL of 0.5 µg/g proposed by FAO^43^ and Egyptian Organization for Standardization and Quality (EOS)^44^ (Table 2; Fig. 4). Meanwhile, 56% (28/50), 41% (41/100), and 22% (22/100) of flathead grey mullet, sardine, and mackerel samples tested, respectively, exceeded the MPL of 2.0 µg/g established by Food Standards Australia New Zealand (FSANZ) for As^45^ (Table 2; Fig. 4). Likewise, 40% of sardine samples from four coastal Egyptian governorates exceeded the Egyptian permissible limit of 2 µg/g for As in fish^3^. Furthermore, 12% and 26% of raw sardine and mackerel samples, respectively, exceeded the MPL for Hg^6^. Meanwhile, only 3.4% (2/60) of Flathead grey mullet from Manzala Lake, Egypt^25^, and 0.16% of 1245 mackerel samples from northern European waters^46^ exceeded the MPL for Hg. Conversely, the majority (80%, 48/60) of the examined Flathead grey mullet had As levels above the maximum recommended levels^25^. On the other hand, all sardine and mackerel samples from retail markets in Bosnia and Herzegovina^47^ and flathead grey mullet from Croatia^48^ contained Hg at concentrations below the MPL, whereas sardine samples from various fish sale markets in Giza, Egypt, were within the MPL for As^49^.

For Pb, 20% (10/50) of flathead grey mullet, 58% (58/100) of sardine, and 45% (45/100) of mackerel samples surpassed the MPL of 0.3 µg/g set by FAO^43^ (Table 2; Fig. 4). The Cd levels in the three fish species studied were lower than the MPL of 0.05 µg/g for Cd in flathead grey mullet and of 0.10 for Cd in sardine and mackerel set by EOS^44^ (Table 2; Fig. 4). Likewise, all sardine and mackerel samples from retail markets in Bosnia and Herzegovina contained Cd concentrations below their MPLs^47^. Furthermore, Cd concentrations were lowest in all fish muscles tested, with only five flathead grey mullet muscle samples exceeding the Cd limits set by Croatia, Germany, and Russia^48^. In contrast, all sardine samples obtained from several fish markets in Giza, Egypt, were higher than the MPL for Cd^49^. Conversely, Abd-Elghany et al. found that 66.6% of flathead grey mullet exceeded the Egyptian MPL for Pb, while 63.4% surpassed the MPL for Cd^25^, whereas Embaby et al.^3^ found that 44% and 50% of sardine samples from four coastal Egyptian governorates exceeded the MPL for Pb and Cd. However, only 10% and 6% of sardine samples and 14% and 10% of mackerel samples exceeded the MPLs for Pb and Cd, respectively^6^. Furthermore, only 0.24% of 1245 mackerel samples from northern European waters exceeded the permissible limits for Cd^46^.

### Comparison of heavy metal concentrations with previous studies

A comparison of the heavy metal concentrations detected in the current study with those previously reported for similar fish species from Egypt and worldwide is presented in Table 3. This comparison highlights the variability in contamination levels across studies, which may be influenced by factors such as geographical locations, fish size and diet, water quality, and other ecological factors.

**Table 3:** Comparison of heavy metal concentrations (µg/g wet weight) in fish samples from the present study with previous studies from Egypt and worldwide.

Fish species	Heavy metals concentrations (µg/g)				Country	References
	Hg	As	Pb	Cd		
Flathead grey mullet	0.313	2.13	0.194	0.018	Egypt	The current study
Sardine	0.131	1.65	0.472	0.021	Egypt	The current study
Mackerel	0.196	0.98	0.37	0.025	Egypt	The current study
Flathead grey mullet	0.198	0.309	0.106	0.052	Croatia	Has-Schön et al.^48^
Flathead grey mullet	0.15	4.25	0.87	0.12	Egypt	Abd-Elghany et al.^25^
Sardine	0.0467	2.645	0.217	0.0125	Spain	Bordajandi et al.^41^
Sardine	0.62	–	2.13	0.55	Algeria	Mehouel et al.^50^
Sardine	0.272	–	0.194	0.0471	Egypt	El-Sherbiny and Sallam ^6^
Sardine	0.089	0.514	0.012	0.020	Bosnia and Herzegovina	Hajrić et al.^47^
Sardine	0.015	6.95	0.008	0.172	Beni-Ensar, Morocco	Kasmi et al.^55^
Sardine	0.068	1.810	0.329	0.03	Egypt	Sabala et al.^7^
Sardine	ND	0.43	0.14	0.34	Egypt	Malak et al.^49^
Mackerel	0.1	0.907	0.0151	0.0126	Spain	Bordajandi et al.^41^
Mackerel	–	3.669	0.003	0.0013	Italy	Copat et al.^53^
Mackerel	0.413	–	0.252	0.0392	Egypt	El-Sherbiny and Sallam ^6^
Mackerel	0.075	0.116	0.005	0.016	Bosnia and Herzegovina	Hajrić et al.^47^
Mackerel	0.046	2.20	ND	0.015	Norway	Frantzen et al.^46^

Guidelines	Hg	As	Pb	Cd	References	
Egyptian Organization for Standardization and Quality (EOS)	0.50	–	0.30	0.05 excluding sardine and Mackerel (0.10)	EOS (No 7136/2010)^44^	
European Commission Regulation (EC)	0.50	–	0.30	0.05 excluding sardine (0.25) and Mackerel (0.10)	EU (No 915/2023)^57^	
FAO	0.50	–	0.30	–	FAO (2018)^43^	
FSANZ	0.50	2.00	0.50	–	FSANZ (2025)^45^	

– Not tested and for Guidelines = No limit established.; ND = Not detected.

In comparison, Has-Schön et al. found a lower mean Hg level of 0.198 ± 0.04 µg/g in the muscles of flathead grey mullet from Croatia^48^, while Bordajandi et al. detected low mean Hg concentrations of 46.73 ng/g (0.0467 µg/g) in sardine and 100 ng/g (0.1 µg/g) in mackerel samples from Huelva, Spain^41^. Similarly, Hajrić et al. found slightly lower Hg levels of 0.075 and 0.089 µg/g in mackerel and sardine samples obtained from the retail market in Bosnia and Herzegovina^47^. Moreover, Abd-Elghany et al. reported lower Hg concentrations in the muscle of flathead grey mullet from Manzala Lake, Egypt, ranging from 0.04 to 0.40 µg/g, with a mean value of 0.15 ± 0.03 µg/g^25^. In contrast, sardine samples from the Algerian coast contained Hg concentrations of 0.620 µg/g^50^, approximately five times higher than that observed in the present study. Conversely, Embaby et al. did not detect Hg in sardine samples collected between 2019 and 2021 from 4 coastal governorates in Egypt (Alexandria, Kafr El-Sheikh, Damietta, and Port Said)^3^.

The current results are comparable to those reported by Sabala et al., who found that the mean As level in raw sardine was 1.810 ± 0.071 µg/g^8^. On the other hand, the highest mean As level in sardines from four Egyptian coastal governorates was 0.475 µg/g^3^, which is lower than those obtained in the present study. Likewise, Has-Schön et al. reported low As levels of 0.309 ± 0.048 µg/g in the muscles of flathead grey mullet from Croatia^48^, while Hajrić et al. observed low As concentrations of 0.116 and 0.514 µg/g in mackerel and sardine, respectively, from retail markets in Bosnia and Herzegovina^47^. In contrast, Abd-Elghany et al. reported higher As levels in the muscles of flathead grey mullet from Manzala Lake, Egypt, ranging from 0.15 to 9.36 µg/g, with a mean value of 4.25 ± 0.47 µg/g^25^. Furthermore, Frantzen et al. analyzed 1245 mackerel (*Scomber scombrus*) samples from northern European waters during 2007–2016 and found that the As concentrations varied between 0.43 and 6.9 µg/g, with a mean value of 2.2 µg/g^46^, which is markedly higher than the mean value of 0.98 µg/g observed in the present study (Fig. 3B).

Our findings agree with those obtained by Embaby et al., who reported mean Pb levels of 0.442 ± 0.016 and 0.482 ± 0.017 µg/g in sardine samples obtained from the Mediterranean coasts of Alexandria and Damietta, respectively, during the winter of 2019^3^. In contrast, Monier et al. obtained a substantially higher Pb concentration of 1.48 ± 0.39 in flathead grey mullet samples obtained from the Damietta fishing port and its surrounding environment in the Mediterranean Sea during winter^7^, while Abd-Elghany et al. detected higher Pb levels in the range of 0.02 to 2.90 with mean values of 0.87 ± 0.15 µg/g in flathead grey mullet from Manzala Lake, Egypt^25^. Conversely, Has-Schön et al. reported slightly lower Pb concentrations of 0.106 ± 0.006 µg/g in the muscle of flathead grey mullet from Croatia^48^. Additionally, El-Sherbiny and Sallam found low mean Pb concentrations of 0.194 ± 0.011 and 0.252 ± 0.025 µg/g in sardine and mackerel samples from the Mediterranean Sea Coast, Egypt^6^, while Hajrić et al. reported very low Pb levels of 0.005 and 0.012 µg/g in mackerel and sardine from retail markets in Bosnia and Herzegovina^47^.

Our findings for Cd were comparable to those reported by Hajrić et al., who found Cd concentrations of 0.020 µg/g in sardine samples from Bosnia and Herzegovina^47^. Meanwhile, Bordajandi et al. found slightly lower mean Cd levels of 12.6 ng/g (0.0126 µg/g) in sardine and 12.5 ng/g (0.0125 µg/g) in mackerel samples from Huelva, Spain^41^. Likewise, Frantzen et al. found that Cd concentration in mackerel samples from northern European waters was 0.015 µg/g^46^. On the other hand, El-Sherbiny and Sallam reported slightly higher mean Cd values of 0.0471 ± 0.003 µg/g and 0.0392 ± 0.003 µg/g in sardine and mackerel, respectively^6^. Furthermore, Has-Schön et al. observed a higher Cd level of 0.052 ± 0.03 µg/g in the muscle tissues of flathead grey mullet from Croatia^48^, while Abd-Elghany et al. observed substantially higher Cd concentrations of 0.12 ± 0.02 µg/g in the muscles of flathead grey mullet from Manzala Lake, Egypt^25^. In contrast, Monier et al. found markedly higher Cd concentrations in flathead grey mullet samples (0.64 ± 0.01 µg/g) and sardine (0.21 ± 0.11 µg/g) collected from the Damietta fishing harbor during the winter season^7^. Similarly, sardine from the Algerian coastline showed considerably higher Cd content of 0.55 µg/g^50^.

### Health risk assessment

The accumulation of HMs in fish muscles may pose a serious health risk to consumers. Previous studies in Egypt^7,25^, China^27^, and Pakistan^51,52^ have evaluated the potential health risks associated with HM consumption by estimating EDI, THQ, TTHQ, and CR.

#### The EDI of HMs detected in fish muscle compared with their PTDI or BMDL values

The EDIs of HMs detected in the muscle of flathead grey mullet, sardine, and mackerel were estimated for both the general population and high-fish consumers and compared to their PTDIs or BMDLs set by regulatory organizations, such as JECFA and EFSA, to assess if the HM concentrations detected in fish samples fall within the safe limit for consumption in humans (Tables 4 and 5). The EDI was calculated based on the average metal concentration in fish muscle and the daily fish consumption rate of 57.09 g/day for the general population^2^and 200 g/day for high-fish consumers^33^ by a 70-kg Egyptian consumer. The EDIs for the general population were lower than PTDIs or BMDLs for the four metals analyzed across all fish species examined, except for Hg in flathead grey mullet, which slightly exceeded the safety threshold (111%) (Table 4). For high-fish-consumption groups, the EDI of Hg in all species, As in flathead grey mullet and sardine, and Pb in sardine and mackerel, exceeded their corresponding PTDI/BMDL values, indicating a potential health concern (Table 5).

**Table 4:** Estimated daily intake (EDI) of heavy metals analyzed in the muscle of flathead grey mullet, sardine, and mackerel compared with their Provisional Tolerable Daily Intakes (PTDI) values or their Benchmark Dose Levels (BMDL) for the general population.

Heavy metals	PTDI/BMDL mg/kg.bw/day	Mean concentration (µg/g)			EDI* (mg of metal /70-kg BW person/day) compared with PTDI or BMDL					
		Flathead grey mullet	Sardine	Mackerel	Flathead grey mullet		Sardine		Mackerel	
					EDI mg/person/d	%EDI/PTDI or BMDL	EDI mg/person/d	%EDI/PTDI or BMDL	EDI mg/person/d	%EDI/PTDI or BMDL
Hg	2.30E−04^a^	0.313	0.131	0.196	2.55E−04	111%	1.07E−04	46.5%	1.59E−04	69.1%
As	3.00E−03^a^	2.13	1.65	0.98	1.74E−03	58%	1.35E−03	45%	7.99E−04	26.6%
Pb	6.30E−04^b^	0.194	0.472	0.37	1.58E−04	25.1%	3.85E−04	61.1%	3.02E−04	47.9%
Cd	8.30E−04^c^	0.018	0.021	0.025	1.47E−05	1.77%	1.71E−05	2.06%	2.04E−05	2.46%

^a^ JECFA^34^; ^b^ EFSA^35^; ^c^ JECFA^36^.

*The estimated daily intake (EDI; mg kg⁻¹ BW day⁻¹) was calculated using the formula: EDI = (Cₘ × IR)/BW, where C_m_ is the concentration of metals in fish samples (mg/kg wet weight), IR is the average daily fish consumption for the general population, and BW is the average body weight.

**Table 5:** Estimated daily intake (EDI) of heavy metals analyzed in the muscle of flathead grey mullet, sardine, and mackerel compared with their Provisional Tolerable Daily Intakes (PTDI) values or their Benchmark Dose Levels (BMDL) for high-fish consumers.

Heavy metals	PTDI/BMDL mg/kg.bw/day	Mean concentration (µg/g)			EDI* (mg of metal /70-kg BW person/day) compared with PTDI or BMDL					
		Flathead grey mullet	Sardine	Mackerel	Flathead grey mullet		Sardine		Mackerel	
					EDI mg/person/d	%EDI/PTDI or BMDL	EDI mg/person/d	%EDI/PTDI or BMDL	EDI mg/person/d	%EDI/PTDI or BMDL
Hg	2.30E−04^a^	0.313	0.131	0.196	8.94E−04	388.7%	3.74E−04	162.6%	5.60E−04	243.5%
As	3.00E−03^a^	2.13	1.65	0.98	6.09E−03	203%	4.71E−03	157%	2.80E−03	93.3%
Pb	6.30E−04^b^	0.194	0.472	0.37	5.54E−04	87.9%	1.35E−03	214.3%	1.057E−03	167.8%
Cd	8.30E−04^c^	0.018	0.021	0.025	5.14E−05	6.19%	6.00E−05	7.23%	7.14E−05	8.60%

^a^ JECFA^34^; ^b^ EFSA^35^; ^c^ JECFA^36^.

*The estimated daily intake (EDI; mg kg⁻¹ BW day⁻¹) was calculated using the formula: EDI = (Cₘ × IR) / BW, where Cₘ is the concentration of metals in fish samples (mg/kg wet weight), IR is the average daily fish consumption for the high-fish consumers, and BW is the average body weight.

For the general population, only the EDI of Hg through consumption of flathead grey mullet exceeded its corresponding PTDI, reaching 111% of the recommended limit. In contrast, the EDIs of As, Pb, and Cd remained below their respective PTDI/BMDL values (Table 4). The current findings align with Monier et al., who reported that the EDIs for Cd and Pb in flathead grey mullet and sardine muscles from the Damietta fishing harbor were lower than the recommended safety thresholds^7^. In contrast, Abd-Elghany et al. reported a lower EDI of 1.3 × 10^−4^ µg/g /d for Hg but higher EDIs of 3.8 × 10^−3^, 7.9 × 10^−4^, and 1 × 10^−4^ µg/g/d for As, Pb, and Cd, respectively, in flathead grey mullet samples^25^. Furthermore, Mehouel et al. estimated the EDIs of Pb and Cd in sardine from Algerian coasts at 1.6 × 10^−5^ and 1.6 × 10^−6^ µg/g bw, respectively^50^, and Copat et al. reported lower EDI values for Pb (1 × 10^−5^ µg/g bw) and Cd (4 × 10^−6^ µg/g bw) in Atlantic mackerel from the Gulf of Catania, Italy^53^, which are considerably lower than the values reported in our study for the general population.

For high-fish consumers, EDIs increased markedly across all fish species. The EDI of Hg exceeded the corresponding PTDI in flathead grey mullet (388.7%), sardine (162.6%), and mackerel (243.5%). Likewise, As exceeded its corresponding BMDL in flathead grey mullet (203%) and sardine (157%), whereas Pb exceeded the recommended limit in sardine (214.3%) and mackerel (167.8%) (Table 5), indicating a potential health concern for high-fish consumers, especially for Hg, As, and Pb. Similarly, the non-carcinogenic EDIs of Hg and As from consuming flathead grey mullet by high fish consumers exceeded their respective PTDIs and BMDLs by 186.33% and 404.76%, respectively^25^. Overall, the higher EDIs of HMs analyzed across fish species examined for both general and high-fish consumers pose a significant public health issue; thus, monitoring fish consumption and adopting strict contamination control measures are crucial to protect public health.

#### THQ and TTHQ

THQ or TTHQ assesses the potential non-carcinogenic health risk linked to exposure to HMs in food^7,25,27^. Values exceeding 1.0 indicate potential non-carcinogenic health risks, whereas values between 0.1 and 1.0 suggest a low health risk, and those < 0.1 denote negligible risk^54^. THQ values for Hg and As in flathead grey mullet, sardine, and mackerel exceeded 1.0 for both the general population and high-fish consumers, indicating a potential non-carcinogenic health concern (Table 6). Whereas values for Pb and Cd were below 1.0 across all fish species, suggesting negligible health risks. Interestingly, THQ values were extremely high among high-fish consumers (Table 6). Overall, the TTHQ values for flathead grey mullet, sardine, and mackerel were 8.4, 5.7, and 4.35 for the general population and 29.43, 19.84, and 15.26 for high-fish consumers, respectively (Table 6).

**Table 6:** Target Hazard Quotient (THQ) and Total Target Hazard Quotients (TTHQ) for assessment of non-carcinogenic health risks of heavy metals associated with the consumption of flathead grey mullet, sardine, and mackerel.

Consumers	Heavy metals	RfD (mg/kg /day)	Flathead grey mullet		Sardine		Mackerel	
			EDI mg/kg BW/day	THQ	EDI mg/kg BW/day	THQ	EDI mg/kg BW/day	THQ
General population	Hg	1E−04^a^	2.55E−04	2.55	1.07E−04	1.07	1.59E−04	1.59
	As	3E−04^a^	1.74E−03	5.8	1.35E−03	4.5	7.99E−04	2.66
	Pb	4E−03^b^	1.58E−04	0.04	3.85E−04	0.09	3.02E−04	0.08
	Cd	1E−03^c^	1.47E−05	0.015	1.71E−05	0.017	2.04E−05	0.02
**Σ HI or TTHQ** ^**d**^				**8.4**		**5.7**		**4.35**
High-fish consumers	Hg	1E−04^a^	8.94E−04	8.94	3.74E−04	3.74	5.60E−04	5.60
	As	3E−04^a^	6.09E−03	20.3	4.71E−03	15.7	2.80E−03	9.33
	Pb	4E−03^b^	5.54E−04	0.14	1.35E−03	0.34	1.057E−03	0.26
	Cd	1E−03^c^	5.14E−05	0.05	6.00E−05	0.06	7.14E−05	0.07
**Σ HI or TTHQ** ^**d**^				**29.43**		**19.84**		**15.26**

RfD: Oral reference dose; EDI: Estimated daily intake; BW: Body weight.

THQ = EDI/RfD according to USEPA (2000)^32^.

^a^ RfD for Hg (MeHg) and As according to USEPA^26^.

^b^ RfD for Pb^37^,^38^.

^c^ RfD for Cd^39^.

^d^ HI (Hazard Index) or TTHQ (Total Target Hazard Quotient) = THQ _Hg_ + THQ _As_+ THQ _Pb_ + THQ _Cd_ USEPA ^26^.

By comparison, none of the THQ or TTHQ values for Cd and Pb in flathead grey mullet or sardine muscles exceeded 1.0^7^. On the other hand, TTHQ values for sardine from the Kafr El-Sheikh governorate surpassed 1.0^3^. Furthermore, the THQ of Hg and the TTHQ values of both Hg and Cd in flathead grey mullet exceeded 1.0 for both the general population and high-fish consumers, indicating a potential non-carcinogenic health risk^25^. Additionally, the THQ values for Cd in *Atlantic mackerel* from the eastern Mediterranean Sea were below 1.0, while values for As were above 1.0, for a consumption rate of more than 1 meal/week, particularly among children^53^. Meanwhile, THQ values for Hg and Cd in sardine and mackerel from Italian supermarkets were < 1.0, indicating a negligible risk. Collectively, TTHQ values for the three fish species examined in the current study exceeded 1, indicating that cumulative exposure poses a substantial non-carcinogenic health concern, primarily due to higher THQ values for both As and Hg (Table 6).

#### Cancer Risk (CR)

CR values between 1 × 10^−6^ (one additional case per 1,000,000 individuals) and 1 × 10^−4^ (one additional case per 10,000 individuals) are not of concern, whereas values exceeding 10^−4^ reveal a potential carcinogenic risk^26^. Arsenic (As) exhibited the highest CR values among the analyzed metals, exceeding 1.0 × 10^−4^ for both the general population and high-fish consumers. CR values for As were 2.61E−03, 2.03E−03, and 1.20E−03 for flathead grey mullet, sardine, and mackerel, respectively, in the general population, while higher values of 9.14E−03, 7.07E−03, and 4.20E−03 were observed for the same fish species in high-fish consumers (Table 7). Meanwhile, CR values of Pb and Cd from consuming flathead grey mullet, sardine, and mackerel were < 1.0 × 10^−4^ for the general population and high fish consumers, indicating a lack of potential carcinogenic risk (Table 7).

**Table 7:** Cancer Risk (CR) associated with heavy metal exposure through consumption of the studied fish species.

Heavy metals	CSF (mg/kg/day)	Flathead grey mullet		Sardine		Mackerel	
		General population	High-fish consumers	General population	High-fish consumers	General population	High-fish consumers
		CR	CR	CR	CR	CR	CR
Hg							
As	1.5^a^	2.61E−03	9.14E−03	2.03E−03	7.07E−03	1.20E−03	4.20E−03
Pb	0.0085^b^	1.34E−06	4.71E−06	3.27E−06	1.15E−05	2.57E−06	8.98E−06
Cd	0.38^b^	5.59E−06	1.95E−05	6.49E−06	2.28E−05	7.75E−06	2.71E−05

$\:CR=CSF\times\:EDI$. CR values between 1E−4 and 1E−6 are acceptable, while values above 1E−4 reveal carcinogenic risk. Cancer risk was calculated according to USEPA^26^.

*No oral Cancer Slope Factor (CSF) has been set for Hg in fish.

^a^ CSF for As was according to USEPA^26^; ^b^ CSF for Pb and Cd were adopted from published literature for Pb and Cd, respectively^8,20,40^.

Similar findings were reported by Abd-Elghany et al., who revealed that CR values of As through consuming flathead grey mullet by both the general population and high-fish consumers were above 1.0 × 10^−4^; in contrast, Pb and Cd values fell within the acceptable thresholds^25^. Likewise, arsenic displayed the highest carcinogenic risk, with a value of 2.5 × 10⁻³ associated with the consumption of sardine samples^8^. Furthermore, Kasmi et al. found that the CR values for As and Cd associated with *Sardina pilchardus* consumption from the Moroccan Mediterranean coast exceeded acceptable limits^55^. Also, CR values for As in most fish species collected from the eastern Mediterranean Sea were above the acceptable lifetime risk threshold^53^. Conversely, Malak et al. found lower CR values of 3.57E−05, 9.44E−07, and 1.02E−04 for As, Pb, and Cd, respectively, in sardine samples from markets in Giza, Egypt^49^.

Overall, the current findings revealed considerable concern regarding As contamination, as CR values of As were above 1.0 × 10^−4^, signifying a potential carcinogenic risk linked to consuming fish contaminated with HMs, especially among high fish consumers. Therefore, it is necessary to monitor HM contamination in fish to ensure food safety and protect public health.

This study has a few limitations that should be considered when interpreting the health risk assessment. First, evaluating health risks based on heavy metal concentrations in raw muscle tissue may overestimate actual human exposure, because cooking processes, such as frying or grilling, can alter metal concentrations through moisture loss and volatilization^56^. Future studies should consider the effects of commonly used cooking methods to improve the accuracy of health risk assessments. Second, chemical speciation of arsenic was not performed, and only total arsenic concentrations were determined. Because marine fish predominantly accumulate less toxic organic arsenic species, such as arsenobetaine, whereas inorganic arsenic represents the primary carcinogenic form, the use of total arsenic concentrations for cancer risk assessment may substantially overestimate the actual carcinogenic risk. Future studies should include arsenic speciation to provide a more accurate assessment of its health risks.

## Conclusion

The present study concluded that flathead grey mullet, sardine, and mackerel samples analyzed contained high concentrations of heavy metals. Arsenic showed the highest levels, whereas Cd exhibited the lowest. Most of the samples examined exceeded permissible limits, particularly for As and Pb. Cd concentrations remained within safe limits across all species. Flathead grey mullet revealed the highest Hg and As concentrations, sardine exhibited the highest Pb levels, and mackerel showed the highest Cd concentration. The EDI of heavy metals through consuming the examined fish species by the general population remained below their PTDIs or BMDLs, except for Hg in flathead grey mullet. However, among high-fish consumers, the intake of Hg in all species, As in flathead grey mullet and sardine, and Pb in sardine and mackerel, exceeded the recommended safety limits, indicating a potential public health concern. In addition, the THQ values for Hg and As, as well as the TTHQ values for the three fish species examined, were above 1.0, indicating a potential non-carcinogenic health risk to the consumer. Furthermore, the carcinogenic risk values of As were higher than 1.0 × 10^−4^ from consuming contaminated fish among both the general population and high-fish consumers, suggesting a potential carcinogenic risk. Hence, continuous monitoring of heavy metal contamination in marketed marine fish, along with stricter regulatory control of pollution sources and raising public awareness of safe consumption practices, is essential to protect public health. Further studies covering a wider range of fish species from different locations in Egypt are crucial to determine the levels of contamination by HMs in fish and to assess the potential health risks.

## Data Availability

All data supporting the findings of this study are included within the article. Any additional information is available from the corresponding author upon reasonable request.
